# Supplementary material for: Neonatal near miss in a State of Northeastern Brazil: Near miss characterization and determinants
Source: PLOS Glob Public Health. 2026 Mar 6;6(3):e0005064. doi: 10.1371/journal.pgph.0005064 (PMC12965687; doi:10.1371/journal.pgph.0005064)
Supplement: Attachment — Submitted filename: Resposta ao Parecer.pdf [file pgph.0005064.s001.pdf]

Dear Editors of *PLOS Global Health*,

We would like to thank you for the careful review of our manuscript entitled **“Neonatal Near Miss in a State of Northeastern Brazil: Near Miss Characterization and Determinants.”** We are also grateful to the reviewers for their constructive and insightful comments, which have contributed significantly to improving our work.

Below, we present our point-by-point responses to the suggestions and comments provided, along with the corresponding modifications made to the manuscript. All changes have been highlighted in the revised version of the text, as requested.

We appreciate the opportunity to submit our study to *PLOS Global Health* and remain at your disposal for any further clarification that may be required.

Sincerely,
Vanessa Moraes Bezerra
On behalf of all authors

Journal Requirements:

1. We have amended your Competing Interest statement to comply with journal style. We kindly ask that you double check the statement and let us know if anything is incorrect.

2. In the online submission form, you indicated that The data is not in the public domain. It was provided by the Secretary of State of Bahia. Therefore, the data will be made available upon reasonable request.

All PLOS journals now require all data underlying the findings described in their manuscript to be freely available to other researchers, either
1. In a public repository,
2. Within the manuscript itself, or
3. Uploaded as supplementary information.

This policy applies to all data except where public deposition would breach compliance with the protocol approved by your research ethics board. If your data cannot be made publicly available for ethical or legal reasons (e.g., public availability would compromise patient privacy), please explain your reasons by return email and your exemption request will be escalated to the editor for approval. Your exemption request will be handled independently and will not hold up the peer review process, but will need to be resolved should your manuscript be accepted for publication. One of the Editorial team will then be in touch if there are any issues.

Answer:

Subject: Justification for Non-Public Availability of Data – Submitted Manuscript
Dear *PLOS Global Health* editorial team,

In response to your request regarding the journal’s data availability policy, we would like to inform you that the data used in our manuscript were provided by the Health Department of the State of Bahia, Brazil (Secretaria da Saúde do Estado da Bahia – SESAB), exclusively for the purposes of this research, following approval by the Research Ethics Committee of the Multidisciplinary Health Institute at the Federal University of Bahia (Approval number: 5.623.213, CAAE: 62056022.9.0000.5556).

These are sensitive data on births and deaths in the state of Bahia, and their unrestricted disclosure could compromise individual confidentiality, even after anonymization. The ethical approval recognizes this risk, and the terms of use established by SESAB restrict access to the research team, without authorization for public availability or unrestricted sharing.

Therefore, we respectfully request an exemption from the requirement of public data availability, as permitted under PLOS policy, due to ethical and legal constraints.
We remain at your disposal for any further information you may require.

3. We noticed that you used “data not shown”/"unpublished data" in the manuscript. We do not allow these references, as the PLOS data access policy requires that all data be either published with the manuscript or made available in a publicly accessible database. Please amend the supplementary material to include the referenced data or remove the references.
Answer: Thank you for your observation, we have made changes to the manuscript to meet the request.

4. Please review your reference list to ensure that it is complete and correct. If you have cited papers that have been retracted, please include the rationale for doing so in the manuscript text, or remove these references and replace them with relevant current references. Any changes to the reference list should be mentioned in the rebuttal letter that accompanies your revised manuscript. If you need to cite a retracted article, indicate the article’s retracted status in the References list and also include a citation and full reference for the retraction notice.

Answer: We reviewed all references included in the manuscript. None of the cited articles have been retracted. All references were checked for completeness and accuracy. No updates or replacements were necessary.

5. Review Comments to the Author
Reviewer #1: Dear authors, I would like to congratulate you on the completion of your article.
The manuscript meets the PLOS Global Public Health criteria for publication. The research is methodologically rigorous, the data support the conclusions, and the study is ethically sound. Statistical analysis was performed appropriately and rigorously. The authors used appropriate statistical tests, and the results are presented clearly and concisely. The manuscript is presented in an understandable manner and written in standard English. However, there are some grammatical and typographical errors that need to be corrected. The study is well written and the results are interesting. However, the authors need to address the following issues:
If possible, make all data underlying the findings available in your manuscript.
Correct any grammatical and typographical errors.

Discuss the public health implications of the results.
Answer:

We appreciate your comments. Regarding the availability of data, we sent an email to the journal discussing the issues of ethical confidentiality and data availability.

We have thoroughly revised the entire manuscript, addressing grammatical, typographical, and stylistic issues to improve clarity and coherence. The language was refined to meet the scientific writing standards expected by PLOS Global Health, with attention to consistency in terminology and alignment with public health and epidemiology discourse. All corrections and improvements are clearly highlighted in the revised manuscript.

We have included more information in the discussion text that reports on the contribution of the research findings to Public Health.

Reviewer #2: Dear Author.

Following are my comments

1. In you manuscript methodology, Neonatal near miss has been defined as any one of the followings: Birth weight < 1500 gm, Gestational age < 32 wks, Apgar score at 5 mins < 7 and Congenital malformation.

Could you explain what are the babies number invidiously.

2. What is the mean birth weight, mean gestational age and mean 5th minute Apgar score.
Answer: We enter the requested information. However, In the database, gestational age is an originally categorized variable, and therefore, it was not possible to calculate mean and standard deviation.
