# Supplementary material for: Neonatal near miss in a State of Northeastern Brazil: Near miss characterization and determinants
Source: PLOS Glob Public Health. 2026 Mar 6;6(3):e0005064. doi: 10.1371/journal.pgph.0005064 (PMC12965687; doi:10.1371/journal.pgph.0005064)
Supplement: Attachment — Submitted filename: Resposta ao parecer.docx [file pgph.0005064.s002.docx]

Dear Editors of *PLOS Global Health*,

We would like to thank you for the careful review of our manuscript entitled **“Neonatal Near Miss in a State of Northeastern Brazil: Near Miss Characterization and Determinants.”** We are also grateful to the reviewers for their constructive and insightful comments, which have contributed significantly to improving our work.

Below, we present our point-by-point responses to the suggestions and comments provided, along with the corresponding modifications made to the manuscript. All changes have been highlighted in the revised version of the text, as requested.

We appreciate the opportunity to submit our study to *PLOS Global Health* and remain at your disposal for any further clarification that may be required.

Sincerely,
Vanessa Moraes Bezerra

On behalf of all authors

Review Comments to the Author

Please use the space provided to explain your answers to the questions above. You may also include additional comments for the author, including concerns about dual publication, research ethics, or publication ethics. (Please upload your review as an attachment if it exceeds 20,000 characters)

Reviewer #1: The manuscript “Neonatal Near Miss in a State of Northeastern Brazil: Characterization and Determinants of Near Miss” (PGPH-D-25-00179R1) showed substantial progress in response to the previous round of review. The authors conducted a thorough review, addressing grammatical, typographical, and stylistic issues, which resulted in a notable improvement in the clarity and coherence of the text. The language was refined to meet scientific writing standards, and there is greater terminological consistency. The methodology is sound, and the statistical analyses appear rigorous and appropriate for the data presented. The findings are relevant to global public health, especially in the Brazilian context.

The remaining changes that warranted a “Minor Revision” are specific and focus on improvements that do not require significant restructuring of the study or additional complex analyses. Points to be addressed include:

Fine terminological consistency: A final passage to ensure absolute consistency of key terms (such as “near miss neonatal” vs. “near miss neonatal”) throughout the manuscript.
Answer: Thank you for this important observation. We carefully reviewed the manuscript and made the necessary adjustments to ensure consistency in the use of the term. We adopted the following standardization: “Neonatal Near Miss” was used in titles and subtitles; “Neonatal near miss” was used at the beginning of sentences; and “neonatal near miss” was used when the term appeared within the body of the text. We believe this adjustment aligns the terminology with scientific writing conventions and improves the overall clarity of the manuscript.

Highlighting public health implications: Although the discussion already addresses the implications, a more concise final paragraph focused on actionable recommendations derived from the results would strengthen the impact of the Discussion section.
Overall, the manuscript is in excellent shape, and the above suggestions are intended only to refine specific aspects to maximize its clarity and full adherence to publication requirements.

Answer: We appreciate the careful and constructive comments. In response to the suggestion regarding the need to better highlight public health implications, we included a concise final paragraph in the Discussion section. This new paragraph synthesizes the main findings and presents actionable recommendations derived from the results, aiming to strengthen the impact and relevance of the study for public health planning and policy formulation. We thank you for the guidance, which helped improve the clarity and completeness of our manuscript.

Reviewer #2: Dear Author,

Thank you for submitting your revision . Now your manuscript looks ok.
